# High Rates of Neutralizing Antibodies to Toscana and Sandfly Fever Sicilian Viruses in Livestock, Kosovo

**DOI:** 10.3201/eid2306.161929

**Published:** 2017-06

**Authors:** Nazli Ayhan, Kurtesh Sherifi, Arber Taraku, Kristaq Bërxholi, Rémi N. Charrel

**Affiliations:** Aix-Marseille University, Marseille, France (N. Ayhan, R.N. Charrel); Fondation Mediterranee Infection Public Hospitals of Marseille, Marseille (N. Ayhan, R.N. Charrel);; University of Hasan Prishtina, Prishtina, Kosovo (K. Sherifi);; Agriculture University of Tirana, Triana, Albania (A. Taraku, K. Bërxholi)

**Keywords:** Bunyaviridae, Phlebovirus, arbovirus, Balkan, Mediterranean, vector-borne infections, viruses, Kosovo, Toscana virus, sandfly fever Sicilian virus, sand flies

## Abstract

Toscana and sandfly fever Sicilian viruses (TOSV and SFSV, respectively), both transmitted by sand flies, are prominent human pathogens in the Old World. Of 1,086 serum samples collected from cattle and sheep during 2013 in various regions of Kosovo (Balkan Peninsula), 4.7% and 53.4% had neutralizing antibodies against TOSV and SFSV, respectively.

Phleboviruses (family *Bunyaviridae*, genus *Phlebovirus*) are negative-sense tri-segmented RNA viruses for which mosquitoes, ticks, and sand flies are vectors. In the Old World, phleboviruses transmitted by sand flies (phlebotomines) are expanding in the Mediterranean basin, where an increasing number of new viruses have been identified (*1*). There, sand fly–borne phleboviruses are divided into 3 groups in accordance with their antigenic relationships. Two groups correspond to recognized species: *Sandfly fever Naples virus* (including sandfly fever Naples [SFNV], Massilia, Tehran, and Toscana [TOSV] viruses) and *Salehabad* virus (including Salehabad and Arbia viruses). The third group comprises 2 viruses classified as tentative species: *Sandfly fever Sicilian virus* (SFSV) and *Corfou virus* (*2*). Several are historic human pathogens, such as SFSV and SFNV, which cause sandfly fever syndrome, a self-limited but severely incapacitating febrile illness (*1*); TOSV can cause central and peripheral nervous system infections, such as meningitis and encephalitis (*3*). 

Although first data on sandfly fever were acquired from the Balkan region, few studies were published specifically about the situation in Kosovo (*4*): in 1976, a total of 9.6% of human serum samples contained neutralizing antibodies against SFSV (*5*) (the exact region of Kosovo was not mentioned), and in 2011, <1% of human serum samples collected in the Pejë region contained TOSV neutralizing antibodies (*6*). Neutralizing antibody–based seroprevalence studies using animal serum proved interesting regarding the global circulation of corresponding viruses, as recently described in Portugal, Tunisia, and Greece (*7**–**9*). To improve understanding of the circulation of TOSV and SFSV in Kosovo, we tested serum samples collected in cattle and sheep through neutralizing assay and field-trapped sand flies for viral RNA.

## The Study

In 2013, serum from domestic animals was collected from 12 different regions of Kosovo. Samples were collected from 933 cattle and 153 sheep from 9 and 5 different regions, respectively, in Kosovo; the information (location, specimen, date) were recorded. Ten milliliters of blood was taken from jugular venipuncture, and serum was separated by centrifugation. All samples were stored at −20°C.

We tested cattle and sheep serum for neutralizing antibodies using the virus microneutralization assay, described for phleboviruses (*7*) in parallel with TOSV strain MRS2010-4319501 and SFSV strain Sabin. Serum samples were diluted from 1:10 to 1:80 into 96-well plates with a volume of 50 μL. Except for controls, we added a 1,000 50% tissue culture infective dose in a 50-μL volume. For controls, we added 50 μL of Eagle's minimum essential medium enriched with 5% fetal bovine serum, 1% penicillin–streptomycin, 1% L-glutamine 200 mmol/L, 1% kanamycin, and 3% fungizone. The plates were incubated at 37°C. After 1 h, a 100 μL suspension of 2 × 10^5^ Vero cells/mL was added and incubated at 37°C in the presence of 5% CO_2_. The microplates were read under an inverted microscope after 5 days for TOSV and 6 days for SFSV, and the presence (neutralization titer at 10, 20, 40, and 80) or absence (no neutralization) of cytopathic effect was noted. Cutoff value for positivity was set at titer >20 (*8*).

In 2014, a total of 267 sand flies were trapped and identified. We tested these sand flies for phleboviruses using previously described protocols (*9*).

Global rates of TOSV neutralizing antibodies were in the same magnitude in cattle (5.14%) and sheep (1.96%) in Kosovo (Table; Figure). Results observed in Pejë were congruent with recent findings obtained with human serum; in both cases, TOSV circulation appears limited (*6*). Although SFNV and TOSV belong to the same serocomplex, they can be distinguished by using neutralization; therefore, the high rate (27.9%) of SFNV neutralizing antibodies in humans reported in the 1970s (*5*) most likely reflects circulation of SFNV rather than TOSV, a finding that did not differ from our results and recent results reported by others (*6*). Rates of TOSV neutralizing antibodies were highest in southwestern regions of Kosovo, whereas negative results were obtained in the Pejë area (Pejë, Deçan, Junik). Although we did not detect viral RNA in the 267 tested sand flies, TOSV was reported in Croatia and Greece (*10**,*), and a new phlebovirus was described in Albania, Croatia, and Bosnia-Herzegovina (*9*; N. Ayhan et al., unpub. data).

**Table:** Neutralizing antibodies against SFSV and TOSV in serum from cattle and sheep, and sandflies trapped in Kosovo, 2013*

Serum source, region	SFSV			TOSV		No. sand flies	Sand fly species (%)
	Total >20	Positive >20, %		Total >20	Positive >20, %		
Cattle, n = 933	546	58.5		48	5.1		
Prizeren, n = 48	20	41.7		5	10.4	75	*Phlebotomus major* (70), *P. simici* (13), *P. tobbi* (8), *P. papatasi* (4), others (5)
Pejë, n = 50	12	24.0		0	0	0	
Rahovec, n = 198	101	51.0		15	7.6	2	*P. major* (100)
Malishevë, n = 165	129	78.2		13	7.9	0	
Glogovac, n = 50	35	70.0		0	0	0	
Klinë, n = 50	39	78.0		5	10.0	0	
Suharekë, n = 245	133	54.3		8	3.3	132	*P. major* (99), P. tobbi (1)
Gjakovë, n = 50	35	70.0		2	4.0	0	
Deçan, n = 77	42	54.6		0	0	0	
Sheep, n = 153	34	22.2		3	2.0		
Junik, n = 28	6	21.4		0	0	58	*P. major* (57), *P. tobbi* (38), others (5)
Hani i Elezit, n = 50	7	14.0		2	4.0	0	
Dragash, n = 30	17	56.7		1	3.3	0	
Pejë, n = 8	3	37.5		0	0	0	
Gjakovë, n = 37	1	2.7		0	0	0	
Total, N = 1,086	580	53.4		51	4.7	267	

*SFSV, sandfly fever Sicilian virus; TOSV, Toscana virus.

**Figure:**
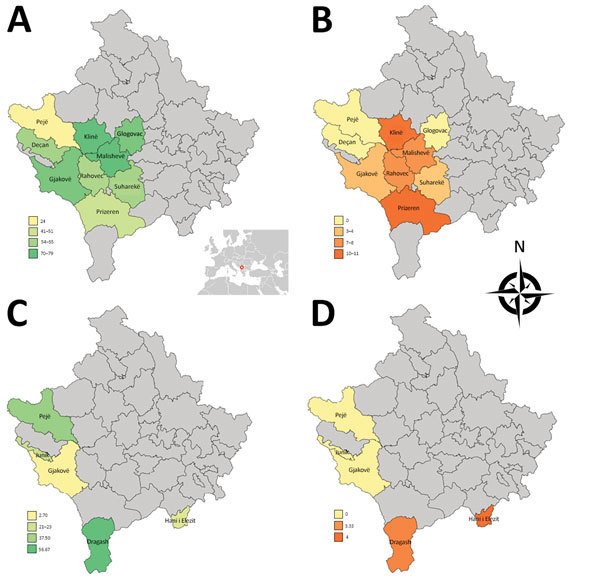
Geographic distribution of rates of neutralizing antibodies against SFSV and TOSV in cattle and sheep, Kosovo, 2013. A) SFSV neutralizing antibodies in cattle. B) TOSV neutralizing antibodies in cattle. C) SFSV neutralizing antibodies in sheep. D) TOSV neutralizing antibodies in sheep. Inset in panel A shows location of Kosovo in Europe. SFSV, sandfly fever Sicilian virus; TOSV, Toscana virus.

Rates of SFSV neutralizing antibodies were much higher than those for TOSV. Results for cattle ranged from 24.0% to 78.2% (mean 58.5%); results for sheep were lower, ranging from 2.7% to 56.7% (mean 22.2%). For sheep, 4 of the 5 regions had rates of 14.0%–56.7%; the rate was much lower (2.7%) in Gjakovë.

Few data are available for comparison; 9.6% of tested human serum contained SFSV neutralizing antibodies in the 1970s (*5*). Although no direct evidence (molecular detection of viral RNA or virus isolation) exists of SFSV or another SFSV-like virus in Kosovo or neighboring countries, our results imply the presence of either SFSV or an SFSV-related virus in Kosovo. We consider it valid to use SFSV as a surrogate for all SFSV-related viruses (sandfly fever Turkey virus, sandfly fever Cyprus virus) because amino acid distances observed between the proteins that elicit neutralizing antibodies (Gn and Gc) are well within the acceptable range, (i.e., <5% different for SFSV and SFSV-related viruses) (*7*). Thus, neutralizing antibodies are unlikely to discriminate between closely related SFSV isolates.

Recent seroprevalence studies showed high seroprevalence rates for SFSV neutralizing antibodies in dogs in Portugal (50.8%) (*7*), Tunisia (38.1%–59.2% depending on the region) (*8*), and Greece (71.9%) and Cyprus (60.2%) (*11**)*. Our results are congruent with data from continental Greece, with rates in the same order of magnitude (*12*). All these findings verify the high prevalence of SFSV in the Mediterranean basin. Because of the nature of this study, the serum was collected and stored under conditions that prevented attempts to detect viral RNA and isolate viral strains. SFSV remains an important human pathogen, as recently highlighted in Africa and Turkey (*12**,**13*).

Published data about the distribution of sand flies and species identification are old and scarce; *Phlebotomus papatasi*, *P. perfiliewi*, *P. neglectus*, and *P. tobbi* sand flies have been documented in Kosovo and neighboring countries with similar environmental and climatic conditions (*14**,**15*). In our study, *P. major* sand flies dominated (81%), followed by *P. tobbi* (11.2%), *P. simici* (3.7%), *P. papatasi* (1%), and other species (3.1%).

SFSV positivity varied among the regions for cattle and sheep. The regional prevalence differences might have resulted from geographic and climatic characteristics of the region that could affect the vector sand fly species distribution and population size.

## Conclusions

Our results confirm that TOSV and SFSV, or an SFSV-like virus, are circulating in several regions of Kosovo, which indicates that humans are exposed to these viruses. This finding merits confirmation through seroprevalence studies and initiation of systematic testing for TOSV and SFSV real-time reverse transcription PCR for febrile illness and central nervous system infections during the warm season.
